# Analysis of drug-induced hearing loss by using a spontaneous reporting system database

**DOI:** 10.1371/journal.pone.0217951

**Published:** 2019-10-08

**Authors:** Mizuki Tanaka, Shiori Hasegawa, Satoshi Nakao, Kazuyo Shimada, Ririka Mukai, Kiyoka Matsumoto, Mitsuhiro Nakamura

**Affiliations:** Laboratory of Drug Informatics, Gifu Pharmaceutical University, Gifu-shi, Gifu, Japan; National Chiao Tung University College of Biological Science and Technology, TAIWAN

## Abstract

Many drugs can cause hearing loss, leading to sensorineural deafness. The aim of this study was to evaluate the risk of drug-induced hearing loss (DIHL) by using the Japanese Adverse Drug Event Report (JADER) database and to obtain profiles of DIHL onset in clinical settings. We relied on the Medical Dictionary for Regulatory Activities preferred terms and standardized queries, and calculated the reporting odds ratios (RORs). Furthermore, we applied multivariate logistic regression analysis, association rule mining, and time-to-onset analysis using Weibull proportional hazard models. Of 534688 reports recorded in the JADER database from April 2004 to June 2018, adverse event signals were detected for platinum compounds, sulfonamides (plain) (loop diuretics), interferons, ribavirin, other aminoglycosides, papillomavirus vaccines, drugs used in erectile dysfunction, vancomycin, erythromycin, and pancuronium by determining RORs. The RORs of other aminoglycosides, other quaternary ammonium compounds, drugs used in erectile dysfunction, and sulfonamides (plain) were 29.4 (22.4–38.6), 18.5 (11.2–30.6), 15.4 (10.6–22.5), and 12.6 (10.0–16.0), respectively. High *lift* score was observed for patients with congenital diaphragmatic hernia treated with pancuronium using association rule mining. The median durations (interquartile range) for DIHL due to platinum compounds, sulfonamides (plain), interferons, antivirals for treatment of hepatitis C virus (HCV) infections, other aminoglycosides, carboxamide derivatives, macrolides, and pneumococcal vaccines were 25.5 (7.5–111.3), 80.5 (4.5–143.0), 64.0 (14.0–132.0), 53.0 (9.0–121.0), 11.0 (3.0–26.8), 1.5 (0.3–11.5), 3.5 (1.3–6.8), and 2.0 (1.0–4.5), respectively. Our results demonstrated potential risks associated with several drugs based on their RORs. We recommend to closely monitor patients treated with aminoglycosides for DIHL for at least two weeks. Moreover, individuals receiving platinum compounds, sulfonamides (plain), interferons, and antivirals for HCV infection therapy should be carefully observed for DIHL for at least several months.

## Introduction

Hearing loss leads to a number of issues such as inability to recognize speech, depression, withdrawal, anger, loss of self-esteem, and poor quality of life (www.healthinaging.org/a-z-topic/hearing-loss). Around 466 million people worldwide have disabling hearing loss, and it is estimated that by 2050, over 900 million people will have disabling hearing loss (www.who.int/news-room/fact-sheets/detail/deafness-and-hearing-loss). Thus, hearing loss can have significant emotional and social impact.

Hearing loss may result from genetic causes, complications at birth, certain viral infections, chronic ear infections, exposure to excessive noise, aging, and ototoxic drugs (www.who.int/news-room/fact-sheets/detail/deafness-and-hearing-loss). More than 150 drugs such as platinum-based anticancer drugs and aminoglycosides are currently known to ototoxic [1]. Ototoxic drugs cause functional impairment and/or cellular degeneration of tissues of the inner ear, and result in sensorineural hearing loss [2]. Since the awareness about drug-induced hearing loss (DIHL) has increased among pharmaceutical companies and healthcare professionals, there is greater knowledge about DIHL. To understand the characteristics of DIHL, the time-to-onset profile of DIHL is important. Cisplatin-induced hearing loss usually starts within days to weeks after treatment, and macrolide-induced hearing loss occurs within 2−7 days after the start of treatment [2]. However, similar information about other ototoxic drugs, it is not well known.

Spontaneous reporting systems (SRSs) such as the Japanese Adverse Drug Event Report (JADER) database of the Pharmaceuticals and Medical Devices Agency (PMDA) has been used in pharmacovigilance assessments. SRSs have served as valuable tools in post-marketing surveillance as they reflect the realities of clinical practice. Several pharmacovigilance indices, such as reporting odds ratio (ROR), have been developed for drug-associated adverse events (AEs) [3]. ROR is a powerful and applicable technique that allows to conduct adjustments through multivariate logistic regression analysis and to control for confounding factors [4–6]. Moreover, association rule mining is a new analytical approach for the discovery of previously undetected relationships, including possible risk factors among variables in huge databases [7–9]. Finally, the time-to-onset analysis using the Weibull shape parameter (WSP) is a useful tool for AE signal detection [6, 10–13]. However, the AE profiles associated with DIHL in the JADER database have not yet been assessed yet.

To the best of our knowledge, our study was the first to evaluate the risk of DIHL associated with prescription drugs by analyzing the JADER database. We estimated DIHL by determining RORs and conducting multivariate logistic regression analysis, association rule mining, and time-to-onset analysis.

## Materials and methods

Information from the JADER database, which included data recorded from April 2004 to June 2018, were obtained from the PMDA website (www.pmda.go.jp). All data from the JADER database were fully anonymized by the regulatory authority before we accessed them. The structure of the JADER database complies with international safety reporting guidelines (International Council for Harmonization of Technical Requirements for Registration of Pharmaceuticals for Human Use [ICH] E2B). The database consists of four data tables: 1) patient demographic information (DEMO), 2) drug information (DRUG), 3) AEs (REAC), and 4) primary illness (HIST). The JADER database does not contain codes for identifying case reports (A1.11), and therefore, we could not exclude duplicate case reports for the same patient (www.pmda.go.jp/files/000145474.pdf). In the DRUG table, the causality of each drug was assigned a code according to its association with the AEs, such as a “suspected drug,” “concomitant drug,” or “interacting drug.” The analysis was restricted to reports in which drugs were recorded as a “suspected drug.”

Age is recorded in the DEMO table that includes patient demographic data. The following age-related items are entered in the DEMO table: < 10, 10–19, 20–29, 30–39, 40–49, 50–59, 60–69, 70–79, 80–89, 90–99, or ≥ 100 years; neonate, baby, infant, child, young adult, adult, or elderly; first trimester, second trimester, or third trimester; and unknown. Reports were stratified by age as follows: ≤ 19, 20–39, 40–59, 60–79, and ≥ 80 years. The ≤ 19 years analysis group included the < 10, 10–19 years, neonate, baby, infant, and child groups. The ≥ 80 years group consisted of the 80–89, 90–99, and ≥ 100 years group. We excluded the items young adult, adult, elderly, first trimester, second trimester, third trimester, and unknown, because these descriptions could not be categorized into precise 20-year intervals.

The AE definitions used in this study corresponded with those in the Medical Dictionary for Regulatory Activities/Japanese version (MedDRA/J, www.pmrj.jp/jmo/php/indexj.php) ver. 19.0. The Standardized MedDRA Queries (SMQ) index consists of groupings of MedDRA terms, ordinarily at the preferred term (PT) level, that relate to a defined medical condition in an area of interest. We used 38 preferred terms for DIHL detection based on the SMQ “hearing disorders” (SMQ code: 20000171) (Table 1). The “hearing disorders” SMQ contains 50 preferred terms. We excluded the following 12 terms that are presumably not related to DIHL: disorders associated with inflammation (acoustic neuritis (PT code: 10063162), hemotympanum (PT code: 10063013), middle ear inflammation (PT code: 10065838), myringitis (PT code: 10061302), myringitis bullous (PT code: 10028659), otosalpingitis (PT code: 10033102), and cholesterol granuloma of the middle ear (PT code: 10008649)); hearing disorders associated with hearing aids (bone anchored hearing aid implantation (PT code: 10070723), cochlea implant (PT code: 10009830), and hearing aid therapy (PT code: 10075385)); hyperacusis (PT code: 10020559); and tinnitus (PT code: 10043882). We used the Anatomical Therapeutic Chemical (ATC) Classification System described by the World Health Organization Collaborating Centre for Drug Statistics Methodology for drug definitions (www.whocc.no/atc_ddd_index/). All generic names of drugs were verified and subsequently linked to the corresponding ATC classification codes. According to the ATC Classification System, drugs related to DIHL were classified into 18 ATC classes (Table 2).

**Table 1:** Preferred terms of “hearing disorders” (SMQ ^a)^ code: 20000171) associated with ototoxic drugs in MedDRA ^b)^.

CODE	Preferred Term	CODE	Preferred Term
10000526	acoustic stimulation tests abnormal (0 case)	10015544	eustachian tube obstruction (1 case)
10075083	altered pitch perception (0 case)	10048865	hypoacusis (182 cases)
10003761	audiogram abnormal (1 case)	10027582	middle ear adhesions (1 case)
10003778	auditory disorder (25 cases)	10062545	middle ear effusion (0 case)
10003789	auditory recruitment (0 case)	10027757	mixed deafness (5 cases)
10048827	autophony (0 case)	10067587	neurosensory hypoacusis (1 case)
10010280	conductive deafness (1 case)	10061327	ossicle disorder (0 case)
10011878	deafness (518 cases)	10063643	otoacoustic emissions test abnormal (0 case)
10052556	deafness bilateral (23 cases)	10033103	otosclerosis (0 case)
10011891	deafness neurosensory (108 cases)	10036626	presbyacusis (1 case)
10011893	deafness occupational (0 case)	10039191	rinne tuning fork test abnormal (0 case)
10011894	deafness permanent (0 case)	10061373	sudden hearing loss (288 cases)
10011900	deafness transitory (7 cases)	10045208	tympanic membrane atrophic (0 case)
10048812	deafness unilateral (43 cases)	10062218	tympanic membrane disorder (1 case)
10013032	diplacusis (0 case)	10045210	tympanic membrane perforation (16 cases)
10049712	dysacusis (5 cases)	10063604	tympanic membrane scarring (0 case)
10014399	electrocochleogram abnormal (0 case)	10045215	tympanometry abnormal (0 case)
10061462	eustachian tube disorder (0 case)	10045218	tympanosclerosis (0 case)
10015543	eustachian tube dysfunction (1 case)	10047878	Weber tuning fork test abnormal (0 case)

^a)^ SMQ: Standardized MedDRA Queries

^b)^ MedDRA: Medical Dictionary for Regulatory Activities

**Table 2:** Number of reports and reporting odds ratio of drug-induced hearing loss ^a)^.

ATC ^b)^ classification	ATC ^b)^ code	Total (n)	Case (n)	ROR ^c)^ (95% CI ^d)^)	Drugs	Total (n)	Case (n)	ROR ^c)^ (95% CI ^d)^)
					Total	534688	1218	
Platinum compounds	L01XA	21661	86	1.8 (1.4−2.2)*	cisplatin	8673	58	3.0 (2.3−4.0)*
					carboplatin	5281	26	2.2 (1.5−3.2)*
					oxaliplatin	8001	8	0.4 (0.2−0.9)
Sulfonamides (plain)	C03CA	2913	77	12.6 (10.0−16.0)*	furosemide	2710	72	12.6 (9.9−16.1)*
					torasemide	247	5	9.1 (3.7−22.1)*
Interferons	L03AB	12785	73	2.6 (2.1−3.3)*	peginterferon alfa-2b	6866	49	3.2 (2.4−4.3)*
					peginterferon alfa-2a	3386	13	1.7 (0.98−2.9)
					interferon alfa-2b	739	4	2.4 (0.9−6.4)
					interferon beta natural	1553	3	0.8 (0.3−2.6)
					interferon alfacon-1	90	2	10.0 (2.5−40.5)*
					interferon alfa natural	354	2	2.5 (0.6−10.0)
Antivirals for treatment	J05AP	13134	67	2.3 (1.8−3.0)*	ribavirin	10394	62	2.7 (2.1−3.5)*
of HCV infections					telaprevir	4257	10	1.0 (0.6−1.9)
					sofosbuvir	1865	6	1.4 (0.6−3.2)
					simeprevir	2285	3	0.6 (0.2−1.8)
					asunaprevir	678	2	1.3 (0.3−5.2)
Other aminoglycosides	J01GB	964	58	29.4 (22.4−38.6)*	gentamicin	264	30	57.5 (39.2−84.5)*
					amikacin	194	11	26.6 (14.4−48.9)*
					kanamycin	49	5	50.0 (19.8−126.2)*
					isepamicin	93	4	19.7 (7.2−53.9)*
					neomycin	85	4	21.7 (7.9−59.3)*
					arbekacin	249	3	5.4 (1.7−16.7)*
					tobramycin	55	2	16.6 (4.0−68.0)*
Protein kinase inhibitors	L01XE	26034	42	0.7 (0.5−0.9)	imatinib	4399	11	1.1 (0.6−2.0)
					sorafenib	4915	10	0.9 (0.5−1.7)
					dasatinib	1255	3	1.0 (0.3−3.3)
					erlotinib	2744	3	0.5 (0.2−1.5)
					axitinib	878	2	1.0 (0.2−4.0)
					lapatinib	731	2	1.2 (0.3−4.8)
					nilotinib	1841	2	0.5 (0.1−1.9)
					osimertinib	653	2	1.3 (0.3−5.4)
					regorafenib	1601	2	0.5 (0.1−2.2)
					ruxolitinib	872	2	1.0 (0.3−4.0)
					dabrafenib	154	1	−^#^
					everolimus	3671	1	−^#^
					gefitinib	2736	1	−^#^
					trametinib	157	1	−^#^
Carcineurin inhibitors	L04AD	16752	38	1.0 (0.7−1.4)	ciclosporin	6602	31	2.1 (1.5−3.0)*
					tacrolimus	10472	7	0.3 (0.1−0.6)
Pyrimidine analogues	L01BC	25158	37	0.6 (0.5−0.9)	tegafur, combinations	8201	16	0.9 (0.5−1.4)
					capecitabine	3561	11	1.4 (0.8−2.5)
					fluorouracil	7796	8	0.4 (0.2−0.9)
					cytarabine	1785	1	−^#^
					gemcitabine	4454	1	−^#^
Taxanes	L01CD	13351	35	1.2 (0.8−1.6)	paclitaxel	6900	25	1.6 (1.1−2.4)*
					docetaxel	5415	11	0.9 (0.5−1.6)
					cabazitaxel	1174	2	0.7 (0.2−3.0)
Monoclonal antibodies	L01XC	25555	32	0.5 (0.4−0.8)	nivolumab	4419	14	1.4 (0.8−2.4)
					trastuzumab	2192	5	1.0 (0.4−2.4)
					bevacizumab	9414	5	0.2 (0.1−0.6)
					cetuximab	2742	2	0.3 (0.1−1.3)
					ipilimumab	545	2	1.6 (0.4−6.5)
					rituximab	3979	2	0.2 (0.1−0.9)
					mogamulizumab	469	1	−^#^
					ofatumumab	83	1	−^#^
					pembrolizumab	2144	1	−^#^
Papillomavirus vaccines	J07BM	2259	32	6.4 (4.5−9.2)*	papillomavirus (human types 16, 18)	1889	23	5.5 (3.6−8.3)*
					papillomavirus (human types 6, 11, 16, 18)	370	9	11.0 (5.7−21.4)*
Drugs used in electile	G04BE	870	29	15.4 (10.6−22.5)*	tadalafil	401	17	19.7 (12.0−32.0)*
dysfunction					sildenafil	478	13	12.4 (7.1−21.5)*
Other antiepileptics	N03AX	10021	27	1.2 (0.8−1.7)	pregabalin	4659	20	1.9 (1.2−3.0)*
					lamotrigine	3446	4	0.5 (0.2−1.4)
					levetiracetam	1866	2	0.5 (0.1−1.9)
					lacosamide	213	1	−^#^
Carboxamide derivatives	N03AF	5568	24	1.9 (1.3−2.9)*	carbamazepine	5568	24	1.9 (1.3−2.9)*
Glycopeptide antibacterials	J01XA	2658	21	3.5 (2.3−5.4)*	vancomycin	1947	20	4.6 (3.0−7.2)*
					teicoplanin	805	3	1.6 (0.5−5.1)
Macrolides	J01FA	5769	20	1.5 (0.98−2.4)	azithromycin	1434	11	3.4 (1.9−6.2)*
					clarithromycin	4066	5	0.5 (0.2−1.3)
					erythromycin	82	2	11.0 (2.7−44.7)*
					spiramycin	2	1	−^#^
					telithromycin	293	1	−^#^
Other quaternary ammonium	M03AC	400	16	18.5 (11.2−30.6)*	pancuronium	30	13	338.5 (164.1−698.5)*
compounds					vecuronium	376	3	3.5 (1.1−11.0)*
Pneumococcal vaccines	J07AL	3011	14	2.1 (1.2−3.5)*	pneumococcus, purified polysaccharides antigen	1457	11	3.4 (1.8−6.1)*
					pneumococcus, purified polysaccharides antigen conjugated	1566	3	0.8 (0.3−2.6)

* The lower limit of 95% CI > 1.

^#^ Number of cases < 2.

^a)^ The number of reports stratified by ATC code or each drug name was calculated.

^b)^ ATC: Anatomical Therapeutic Chemical

^c)^ ROR: reporting odds ratio

^d)^ CI: confidence interval

**Fig 1:**
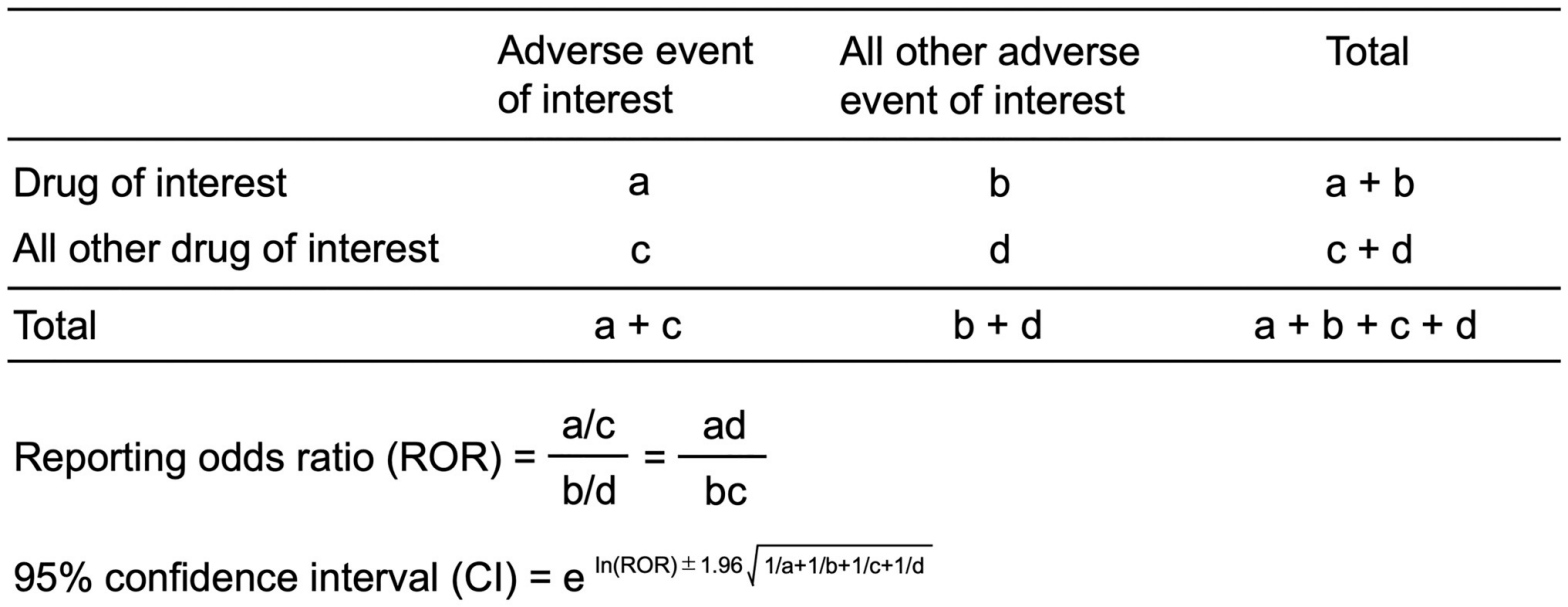
Two by two contingency table for analysis.

The authorized pharmacovigilance index ROR [3] was calculated using a two-by-two contingency table pertaining to the presence or absence of a particular drug and AE in the case reports. ROR is the ratio of odds of reporting an AE (DIHL-related AE) versus all other events associated with the given drug compared to the reporting odds for all other drugs in the JADER database (Fig 1) [3]. ROR was expressed as a point estimate with a 95% confidence interval (CI) [4]. Signals were considered statistically significant, if the lower limit of the 95% CI was above 1; at least two cases were required for analysis [14].

We refined the ROR signal with a dedicated correction to detect possible confounders in the database [15]. Furthermore, ROR was adjusted using a multivariate logistic regression model. To calculate the adjusted ROR, only reports with complete information regarding reporting year, sex, and age were extracted from the JADER database. The following formula was used:

$$\text{log}\left(odds\right)=\beta_{0}+\beta_{1}Y+\beta_{2}S+\beta_{3}A+\beta_{4}S*A$$

Reporting year (Y), sex (S), and the age-stratified group (≤ 19 years, 20–39 years, 40–59 years, 60–79 years, and ≥ 80 years) (A) were defined as independent variables. The dependent variable was a binary response to the absence or presence of DIHL in each report. To comparatively evaluate the effect of factors, we selected explanatory variables using a stepwise method [16, 17] and a significance level of 0.05 (forward and backward). The contribution of selected variables in the final model was evaluated. A likelihood ratio test was used to assess the influence of explanatory variables.

A mosaic plot of the contingency table was constructed using the age-category (X) and ATC classification of DIHL-related drugs (Y). The proportions on the x-axis represent the number of observations for each level of the X variable. The mosaic plot is divided into rectangles, and the vertical length of a rectangle is proportional to the size of the Y variable at each level of the X variable.

We evaluated drug dose-dependency using patient demographic data (DEMO) and drug information (DRUG). We estimated the daily dose utilizing the daily dose per square meter (mg/m^2^) based on body weight and height against each case in the JADER database. Next, we compared the doses of suspected drugs between patients with and without DIHL using Student’s *t*-test.

Association rule mining is a common technique used to identify associations among numerous variables. Given a set of transactions ***T*** (each transaction is a set of items), an association rule can be expressed as X [lhs: left-hand-side, the antecedent of the rule] → Y [rhs: right-hand-side, the consequent of the rule], where X and Y are mutually exclusive sets of items [18]. If the association rule of X → Y is true, then *support*, *confidence*, and *lift* values can be calculated to evaluate the correlation of this rule. S*upport* determines how often a rule, which in this case is the combination of X and Y, is observed in the database. *Support* was measured using the following formula:

Support=P(X∩Y)={X∩Y}/{D},

where D is the total number of transactions in the database.

The *confidence* of the association rule demonstrates the rule’s strength [19]. *Confidence* was calculated by the following equation:

Confidence=P(X∩Y)/P(X)

*Lift* represents the ratio of probability. Given a rule, X and Y occur together to the multiple of the two individual probabilities for X and Y:

Lift=P(X∩Y)/P(X)P(Y)

*Lift* evaluates the independence of X and Y, and higher *lift* values indicate stronger relationships. If X and Y are independent, *lift* equals 1. If X and Y are positively or negatively correlated, *lift* is > 1 or < 1, respectively. Association rule mining was performed utilizing the *arules* package of the R software (version 3.6.0). The parameter *maxlen* (maximum length of itemset/rule, a parameter in the *arules* package) is the maximum size of mined frequent itemsets. To extract association rules efficiently, the thresholds for the optimized *support*, *confidence*, and *maxlen* are defined depending on factors such as size of the data, number of items, and research purpose. In this study, we defined the minimum *support* and *confidence* thresholds as 0.00001 and 0.1, respectively; furthermore, *maxlen* was restricted to 3.

Time-to-onset duration from the JADER database was calculated from the time of the patient’s first prescription to the occurrence of the AEs. The median duration, quartiles, and WSPs were used to evaluate the time-to-onset data. The scale parameter α of the Weibull distribution determines the scale of the distribution function. A larger-scale value (α) stretches the distribution, while a smaller scale value shrinks the data distribution. The shape parameter β of the Weibull distribution determines the shape of the distribution function. A larger shape value gives a left-skewed curve, whereas a smaller shape value gives a right-skewed curve. In the analysis of the SRS, the shape parameter β of the Weibull distribution was used to indicate hazards without a reference population as follows. When β was equal to 1, the hazard was estimated to be constant over time. If β was greater than 1 and the 95% CI of β excluded the value 1, the hazard was considered to increase over time. Finally, if β was less than 1 and the 95% CI of β excluded the value 1, the hazard was considered to decrease over time [6, 10–13]. Data analyses were performed using JMP, version 12.0.1 (SAS Institute Inc., Cary, NC, USA).

## Results

The JADER database contains 534688 reports submitted from April 2004 to June 2018, and we identified 1218 DIHL events. The top seven AEs were deafness (518 cases, PT code: 10011878), sudden hearing loss (288 cases, PT code: 10061373), hypoacusis (182 cases, PT code: 10048865), deafness neurosensory (108 cases, PT code: 10011891), deafness unilateral (43 cases, PT code: 10048812), auditory disorder (25 cases, PT code: 10003778), and deafness bilateral (23 cases, PT code: 10052556) (Table 1). The lower limits of 95% CI of RORs for platinum compounds, sulfonamides (plain), interferons, antivirals for treatment of HCV infections, other aminoglycosides, papillomavirus vaccines, drugs used in erectile dysfunction, carboxamide derivatives, glycopeptide antibacterials, other quaternary ammonium compounds, and pneumococcal vaccines were over 1. The RORs for other aminoglycosides, other quaternary ammonium compounds, drugs used in erectile dysfunction, and sulfonamides (plain) were 29.4 (22.4–38.6), 18.5 (11.2–30.6), 15.4 (10.6–22.5), and 12.6 (10.0–16.0), respectively (Table 2). The drugs for which the lower limits of 95% CI of RORs were over 1 and RORs were over 10 were as follows: other quaternary ammonium compounds (pancuronium), other aminoglycosides (gentamicin, amikacin, kanamycin, isepamicin, neomycin, tobramycin), drugs used in erectile dysfunction (sildenafil, tadalafil), for sulfonamides (plain) (furosemide), papillomavirus vaccines (human types 6, 11, 16, 18), and macrolides (erythromycin) (Table 2).

**Fig 2:**
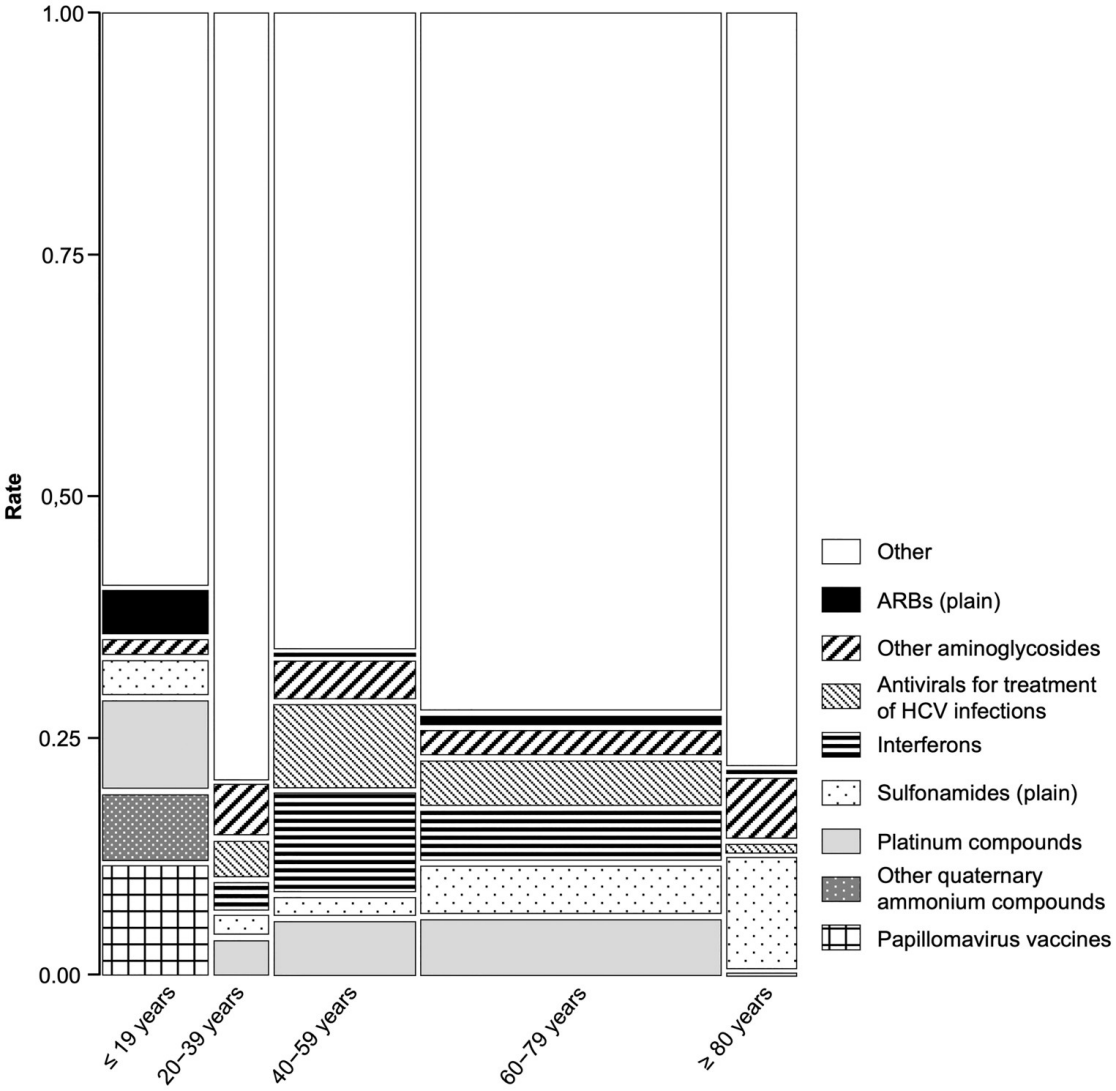
Mosaic plot of drug-induced hearing loss.

Using a stepwise logistic regression model, we examined and selected significant DIHL-related variables among the demographic factors (sex and age-stratified group) and reporting year. The results in the final model indicated significant contributions to DIHL of the ≤ 19 years group (p < 0.0001), the female and ≤ 19 years group (p < 0.0001), and the female and 40–59 years group (p = 0.0012). Contrarily, the reporting year did not significantly contribute to DIHL (data not shown). Adjusted RORs for the analyzed groups were as follows: 1.10 for the female group (0.97–1.25, reference: control male group); 2.03 for the ≤ 19 years group (1.68–2.47, reference: ≥ 20 years group); 1.13 for the 40–59 years group (0.97–1.32, reference: ≤ 39 or ≥ 60 years group); 4.32 for the female and ≤ 19 years group (2.94–6.34, reference: male or other age female group); and 0.75 for the female and 40–59 years group (0.55–1.03, reference: male or other age female group). In the mosaic plot, papillomavirus vaccines or other quaternary ammonium compounds were associated with DIHL only in the ≤ 19 years group, causing DIHL in 12.0% and 7.4% of the cases, respectively (Fig 2).

Furosemide, ribavirin, and cisplatin were the three drugs most frequently associated with DIHL (Table 2). Therefore, we investigated the dose-dependency of these drugs. The average doses (mean ± standard deviation) of furosemide, ribavirin, and cisplatin for cases with DIHL and without DIHL was 43.7 ± 13.5 and 43.7 ± 2.3 mg (p = 0.9990); 696.2 ± 27.3 and 657.5 ± 2.1 mg (p = 0.1569); and 106.0 ± 12.9 and 88.9 ± 1.0 mg (p = 0.1873), respectively.

**Fig 3:**
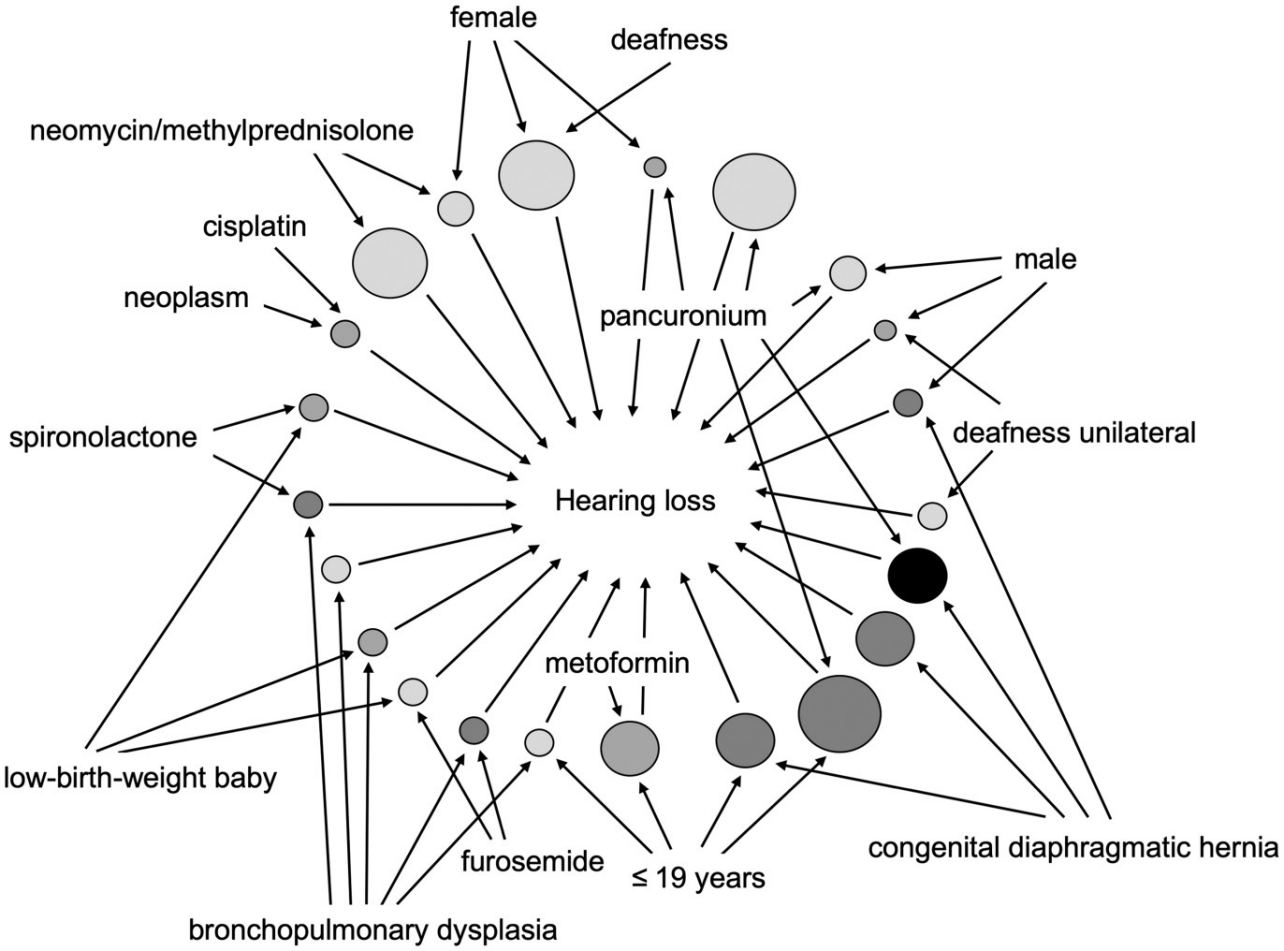
Association rules for drug-induced hearing loss based on the JADER database between April 2004 and June 2018. *Support* and *lift* were presented using the R-extension package *arulesViz* which implements novel visualization techniques to explore association rules. The plot arguments in the *arulesViz* were set as follows: method = “graph,” measure = “*support*,” and shading = “*lift*.” The *support* measures were visualized by the size of the circle area. The *lift* measures were presented by the color shade of the circles.

Next, we evaluated the possible association between DIHL and demographic data. The mining algorithm identified a set of 22 rules for DIHL (Table 3). {Pancuronium, congenital diaphragmatic hernia} → {hearing loss} demonstrated the highest *lift* value (Table 3 (id [1]), Fig 3). The association rules {≤ 19 years, pancuronium} → {hearing loss}, {≤ 19 years, congenital diaphragmatic hernia} → {hearing loss}, and {congenital diaphragmatic hernia} → {hearing loss} exhibited high scores for *lift* and *support* (Table 3 (id [5]–[7]), Fig 3).

**Table 3:** Association rule mining for drug-induced hearing loss (sorted by *lift*).

id	lhs ^a)^		rhs ^b)^	*support*	*confidence*	*lift*
[1]	{pancuronium, congenital diaphragmatic hernia}	→	{hearing loss}	2.1E-05	1.00	444.46
[2]	{congenital diaphragmatic hernia, male}	→	{hearing loss}	1.3E-05	0.47	207.42
[3]	{furosemide, bronchopulmonary dysplasia}	→	{hearing loss}	1.3E-05	0.47	207.42
[4]	{spironolactone, bronchopulmonary dysplasia}	→	{hearing loss}	1.3E-05	0.44	194.45
[5]	{≤ 19 years, pancuronium}	→	{hearing loss}	2.6E-05	0.42	188.56
[6]	{≤ 19 years, congenital diaphragmatic hernia}	→	{hearing loss}	2.1E-05	0.42	188.04
[7]	{congenital diaphragmatic hernia}	→	{hearing loss}	2.1E-05	0.41	181.08
[8]	{bronchopulmonary dysplasia, low-birth-weight baby}	→	{hearing loss}	1.3E-05	0.32	141.42
[9]	{pancuronium, female}	→	{hearing loss}	1.1E-05	0.30	133.34
[10]	{spironolactone, low-birth-weight baby}	→	{hearing loss}	1.3E-05	0.29	129.63
[11]	{cisplatin, neoplasm}	→	{hearing loss}	1.3E-05	0.28	124.45
[12]	{male, deafness unilateral}	→	{hearing loss}	1.1E-05	0.25	111.12
[13]	{≤ 19 years, metoformin}	→	{hearing loss}	2.1E-05	0.24	106.28
[14]	{furosemide, low-birth-weight baby}	→	{hearing loss}	1.3E-05	0.19	84.09
[15]	{deafness unilateral}	→	{hearing loss}	1.3E-05	0.18	77.78
[16]	{pancuronium}	→	{hearing loss}	2.6E-05	0.16	71.52
[17]	{≤ 19 years, bronchopulmonary dysplasia}	→	{hearing loss}	1.3E-05	0.15	64.82
[18]	{bronchopulmonary dysplasia}	→	{hearing loss}	1.3E-05	0.14	62.22
[19]	{neomycin/methylpredonisolone}	→	{hearing loss}	2.4E-05	0.14	60.19
[20]	{neomycin/methylpredonisolone, female}	→	{hearing loss}	1.5E-05	0.13	59.26
[21]	{pancuronium, male}	→	{hearing loss}	1.5E-05	0.12	53.07
[22]	{female, deafness}	→	{hearing loss}	2.4E-05	0.10	45.50

^a)^ lhs: left-hand side (antecedents)

^b)^ rhs: right-hand side (consequents)

**Fig 4:**
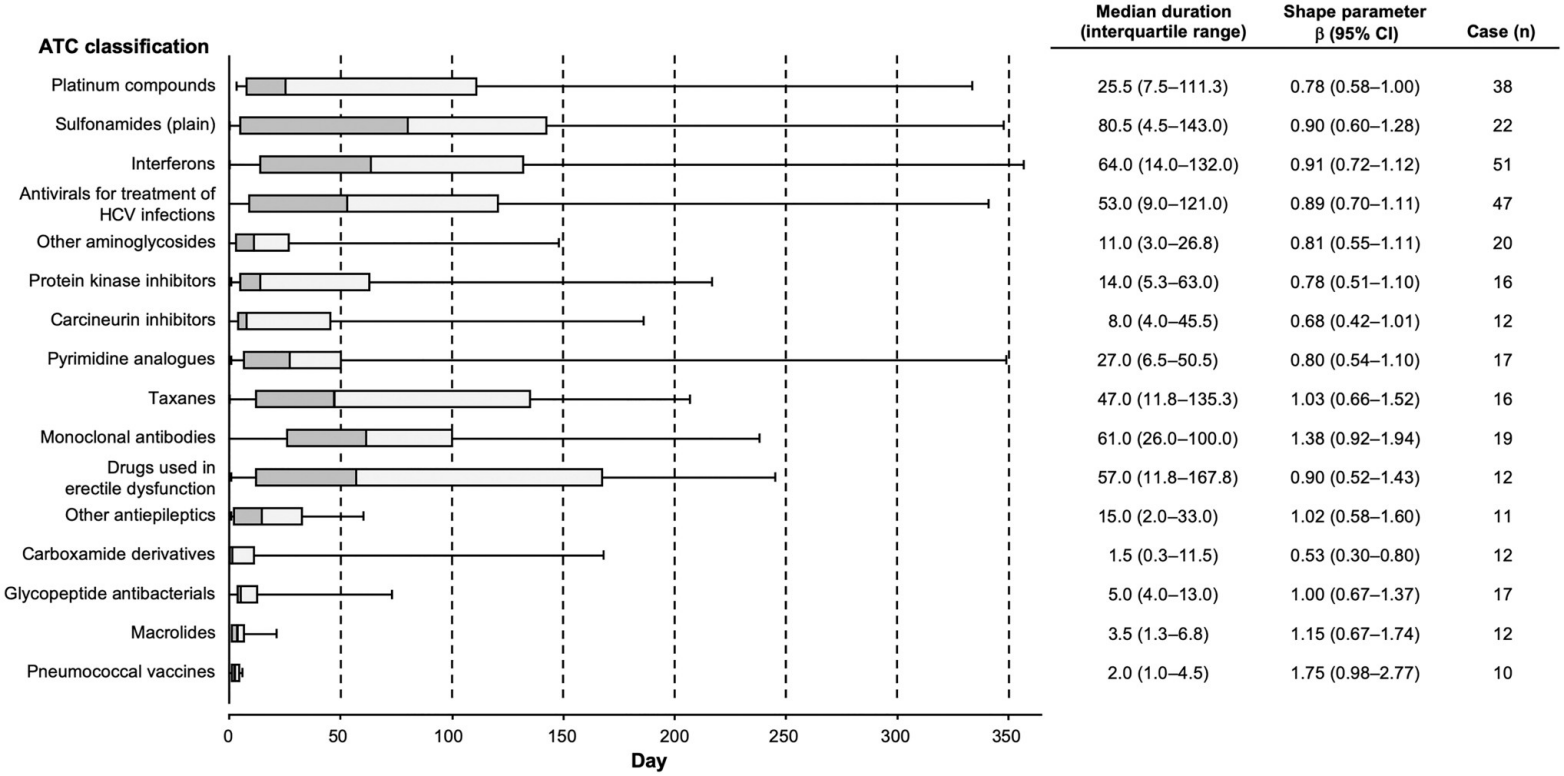
A box plot and the Weibull shape parameter of drug-induced hearing loss for each ATC classification of drugs.

For the time-to-onset analysis, we extracted combinations for which complete information with regard to the date of treatment initiation and the date of AE onset were available. We evaluated 16 ATC classifications of drugs for which the number of reported cases was more than 10 (Fig 4). The median durations (interquartile range) for DIHL due to platinum compounds, sulfonamides (plain), interferons, antivirals for treatment of HCV infections, other aminoglycosides, drugs used in erectile dysfunction, carboxamide derivatives, glycopeptide antibacterials, macrolides, and pneumococcal vaccines were 25.5 (7.5–111.3), 80.5 (4.5–143.0), 64.0 (14.0–132.0), 53.0 (9.0–121.0), 11.0 (3.0–26.8), 57.0 (11.8–167.8), 1.5 (0.3–11.5), 5.0 (4.0–13.0), 3.5 (1.3–6.8), and 2.0 (1.0–4.5), respectively. The observed ratios for DIHL due to platinum compounds, sulfonamides (plain), and other aminoglycosides within the first 4 weeks after administration were 51.3% (20/39), 41.7% (10/24), and 80.0% (16/20), respectively. The upper limits of the 95% CI of the WSP β value for carboxamide derivatives were less than 1.

## Discussion

Our results suggest that AE signals for DIHL were detected for several drugs in the JADER databases. The risks of DIHL due to platinum compounds, sulfonamides (plain), interferons, antivirals for treatment of HCV infections, other aminoglycosides, drugs used in erectile dysfunction, glycopeptide antibacterials, and other quaternary ammonium compounds are stated in the many reports [20–37], which agrees with our results. On the other hand, the ROR signals of protein kinase inhibitor, pyrimidine analogues, and monoclonal antibodies were not detected.

Aging is a risk factor for hearing loss [38, 39]. However, multivariate logistic regression analysis indicated that the ≤ 19 years group and the female and ≤ 19 years group may be risk factors for DIHL in our study. To clarify the cause of this discrepancy, we compared the ATC classification types of the drugs administered among the age groups. Other quaternary ammonium compounds (pancuronium and vecuronium) caused DIHL only in the ≤ 19 years group (7.4%) (Fig 2). In association rule mining, the *lift* and *support* values of {≤ 19 years, pancuronium} → {hearing loss} were high (Table 3 (id [5]), Fig 3). Therefore, using pancuronium may largely contribute to DIHL in the ≤ 19 years group. Moreover, papillomavirus vaccines were associated with DIHL only in the ≤ 19 years group (12.0%) (Fig 2) and the female group, implying their high contribution to DIHL in these patient populations. Our analysis suggested an association between ≤ 19 years age and DIHL. However, through the analysis of standardized patient background based on data subsetting and multivariate logistic regression mitigating the influence of confounding factors [4–6, 15–17, 40], we may be able to detect the effects of aging. Male sex is a risk factor for hearing loss [38, 39]. However, in the multivariate logistic regression analysis sex was not related to DIHL. This finding may be influenced by different drugs being used in males and females (e.g. papillomavirus vaccines).

Next, we investigated the dose-dependency of furosemide, ribavirin, and cisplatin effects. The p values for ribavirin (p = 0.1569) and cisplatin (p = 0.1873) displayed a tendency of being low. However, there were no significant differences in the average doses between cases with and without DIHL.

Time-to-onset profiles of DIHL have been systematically addressed using the JADER database. Our results suggest that attention should be paid to the possibility of DIHL onset with aminoglycosides and careful observation is recommended soon after the administration for at least 2 weeks. The median durations for DIHL due to platinum compounds and macrolides were 25.5 and 3.5, respectively, and these results support those reported previously [2]. The median duration for DIHL due to macrolides and pneumococcal vaccines was a few days and the interquartile range was less than a week (Fig 4). The median durations for DIHL due to carboxamide derivatives, i.e., carbamazepine, was a few days. The upper limit of the 95% CI of the shape parameter β value for carboxamide derivatives was less than 1, and the hazard was considered to decrease over time (Fig 4). Therefore, after treatment with macrolides, pneumococcal vaccines, and carbamazepine, it may be necessary for a few days to pay attention to whether hearing loss occurs. On the other hand, special attention for a prolonged duration should be paid to the possibility of DIHL onset due to platinum compounds, sulfonamides (plain), interferons, and antivirals for treatment of HCV infections, and careful observation for at least several months is recommended after the administration of these drugs.

Cisplatin chemotherapy has been widely used in cancer treatment [20]. Following cisplatin chemotherapy, the incidence of hearing loss is high and 60% of affected children develop permanent hearing loss [20]. The related ROR signal was detected in our study.

It is reported that high trough levels for aminoglycosides (such as gentamicin, tobramycin, and amikacin treatment) and aging may be the risk factors for auditory toxicity [22]. Neomycin is considered most ototoxic, followed by gentamicin, kanamycin, and tobramycin while amikacin and netilmicin are considered the least ototoxic [23]. Our analysis showed the lower limits of 95% CI of RORs value were >1 with gentamicin, kanamycin, amikacin, neomycin, isepamicin, tobramycin, and arbekacin, and there are no reports of DIHL due to netilmicin.

The ROR signals were detected for sulfonamides (plain) and vancomycin (Table 2). It is said that high doses of loop diuretics such as furosemide and vancomycin cause ototoxicity [24, 25]. Furthermore, co-administration of loop diuretics or vancomycin can increase aminoglycoside-induced ototoxicity [26]. Patients who were administered only sulfonamides (plain) (loop diuretics), only vancomycin, or only other aminoglycosides accounted for 98.7% (76/77 cases), 90.0% (18/20 cases), 94.8% (55/58 cases) of the cases, respectively. In this study, DIHL was observed in most patients who were administered only loop diuretics or only vancomycin. Thus, patients treated with only loop diuretics or only vancomycin should be closely monitored for DIHL.

Several cases of hearing loss during interferon therapy or combination treatment with peginterferon and antiviral for HCV infections (ribavirin) have been reported [27–30]. In our study, interferons and ribavirin were coadminstered in 78.1% (57/73 cases) of patients with hearing loss, and 21.9% (16/73 cases) patients were administered interferons alone. Five patients who received ribavirin and not take interferons showed hearing loss. Further studies are necessary on whether interferons themselves are ototoxic and whether coadministration of interferons and ribavirin increases ototoxicity associated with interferons.

ROR signals were detected for papillomavirus vaccines (Table 2). To the best of our knowledge, reports of the association between papillomavirus vaccines and hearing loss are rare. However, cognitive decline is as a frequent papillomavirus vaccine AE [41]. Furthermore, hearing loss has been associated with cognitive decline, which improves with the use of hearing aids [42, 43]. Our analysis demonstrated cognitive decline in 59.4% (19/32 cases) of the patients with papillomavirus vaccine-related hearing loss. Thus, hearing loss related to papillomavirus vaccines may be partially associated with cognitive decline.

Phosphodiesterase 5 (PDE5) inhibitors such as sildenafil are prescribed for erectile dysfunction [44]. Case studies and retrospective chart reviews have suggested that PDE5 inhibitors may induce sensorineural hearing loss [31–33]. On the contrary, sildenafil was reported to not have any effect on hearing in a mouse model [44]. The pathophysiology and mechanism of PDE5 inhibitor-induced hearing loss are not yet well known. Our analysis showed that significant ROR signals were detected with drugs used in erectile dysfunction, and the lower limits of 95% CI of RORs value were >1 with tadalafil and sildenafil (Table 2). This may indicate that PDE5 inhibitors are a risk factor of DIHL. Erectile dysfunction may be associated with age [45]. The ototoxicity associated with PDE5 inhibitors may enhance age-related hearing loss.

Sensorineural hearing loss occurs in up to 60% of survivors of neonatal congenital diaphragmatic hernia [35]. Furthermore, prolonged pancuronium use may be associated with sensorineural hearing loss in patients with congenital diaphragmatic hernia [35, 36]. Contrarily, in congenital diaphragmatic hernia patients, only age independent association of sensorineural hearing loss has been reported, which did not involve longer aminoglycosides, furosemide, and pancuronium treatments [46]. Our analysis showed that hearing loss occurred in 43.3% of the patients (13/30 cases) with AEs due to pancuronium, and the ROR signal was detected (Table 2). The association rule {pancuronium, congenital diaphragmatic hernia} → {hearing loss} demonstrated the highest *lift* value (Table 3 (id [1]), Fig 3): Furthermore, {≤ 19 years, pancuronium} → {hearing loss}, {≤ 19 years, congenital diaphragmatic hernia} → {hearing loss}, and {congenital diaphragmatic hernia} → {hearing loss} exhibited high *lift* and *support* scores (Table 3 (id [5–7]), Fig 3). Therefore, not only age, but also pancuronium administration may be risk factors for sensorineural hearing loss in patients with congenital diaphragmatic hernia, highlighting the need for monitoring after pancuronium treatment in this patient group.

In the JADER database, duplicate data may be present due to follow-up reports on a case or different individuals disclosing the same patient case. It is recommended to identify duplicate patient reports originating from different sources and to exclude them from the analysis. For example, for the US Food and Drug Administration (FDA) Adverse Event Reporting System (FAERS), we followed the agency’s recommendations (www.fda.gov/Drugs/GuidanceComplianceRegulatoryInformation/Surveillance/AdverseDrugEffects) and adopted the most recent case number to identify duplicate patient reports and exclude them from the analysis. However, there is no key code to identify duplicate reports in the JADER database, rendering their removal challenging. The PMDA has introduced an evaluation method based on a match score for duplicate detection in the JADER database [47]. However, this analytical approach is currently not widely accepted, and most JADER database reports do not distinguish between duplicates. Therefore, we did not further investigate this topic in the current study.

SRSs are subject to over-reporting, under-reporting, missing data, exclusion of healthy individuals, lack of denominators, and presence of confounding factors [3]. The JADER database is an SRS and does not contain sufficient information regarding patient background to allow proper evaluation. Furthermore, many factors influence DIHL. Thus, increased reactive oxygen species contribute to cisplatin-induced hearing loss [21]. Viral infections result in hearing loss by direct damage to inner ear structures induction inflammatory responses, and increased susceptibility to bacterial or fungal infections [48]. Noise induces synaptic loss between inner hair cells and spiral ganglion neurons and may be a key pathological factor for sensorineural hearing loss [49]. The effects of these factors on DIHL are difficult to evaluate by using the SRS data set. Future investigations should conduct more detailed analysis focused on DIHL.

Despite the limitations inherent to spontaneous reporting, risk factors for DIHL were identified after adjustment for patient differences using appropriate analysis methods. Our results, based on the evaluation of JADER, are consistent with previous reports and provide essential information to improve our understanding of this issue. Furthermore, our study indicates the importance of comparing safety profiles of newer and traditional drugs using post-marketing real-world data. Information from the JADER dataset may be considered of complementary value. However, these data may be particularly beneficial to prescribers.

## Conclusions

To best of our knowledge, this was the first study to evaluate DIHL and the responsible drugs using an SRS analysis strategy. Based on RORs, we demonstrated the potential DIHL risk associated with numerous drugs including platinum compounds, sulfonamides (plain) (loop diuretics), interferons, ribavirin, other aminoglycosides, papillomavirus vaccines, drugs used in erectile dysfunction, vancomycin, erythromycin, and pancuronium. Our finding indicated that patients with congenital diaphragmatic hernia should be monitored after pancuronium administration. Patients treated with aminoglycosides should be closely observed for DIHL for at least 2 weeks. After the administration of macrolides, pneumococcal vaccines, and carbamazepine, it may be necessary to monitor individuals for hearing loss for a few days. However, careful observation for at least several months is recommended after the administration of platinum compounds, sulfonamides (plain), interferons, and antivirals for treatment of HCV infections. Despite the inherent limitations associated with SRS data, we believe that our findings represent a valuable contribution to the clinical knowledge and will help improve the management of DIHL.
